# Hon. Roland Ellis of Macon

**Published:** 1902-04

**Authors:** 


### HON. ROLAND ELLIS OF MACON.

MR. ROLAND ELLIS, of Bibb County, will offer for election to the House of Representatives, and if returned will probably be a candidate for speaker. In recognition of Mr. Ellis' services to the medical profession by his energetic, able and successful resistance to the passage of the "Osteopathy Bill" by the Senate in 1900, it is hoped that every member of the profession will use his influence with the representatives from his county in Mr. Ellis' behalf. The defeat of this obnoxious measure was largely due to the efforts of Mr. Ellis, and his valuable assistance at a critical time should not go unnoticed.

